# A Case of Surgery for a Giant Popliteal Venous Aneurysm Positive for Heparin-Induced Thrombocytopenia Antibodies with Repeated Acute Pulmonary Embolism

**DOI:** 10.3400/avd.cr.25-00031

**Published:** 2025-06-17

**Authors:** Satoru Tomita, Koki Yokawa, Kazufumi Yoshida, Kenta Masada, Yosuke Inoue, Yoshimasa Seike, Hitoshi Matsuda

**Affiliations:** Department of Cardiovascular Surgery, National Cerebral and Cardiovascular Center, Suita, Osaka, Japan

**Keywords:** popliteal venous aneurysm, heparin-induced thrombocytopenia, pulmonary embolism

## Abstract

A 56-year-old woman with recurrent acute pulmonary embolism was diagnosed with a left popliteal venous aneurysm (PVA) by venous echocardiography. During anticoagulation therapy with heparin for acute pulmonary embolism, she was found to be positive for heparin-induced thrombocytopenia (HIT) antibodies. Surgery was performed with argatroban for anticoagulation, removal of the thrombus in the PVA, and suturing of the vein. Postoperatively, the popliteal vein showed shrinkage, and no recurrence of thromboembolism was observed. We report a case in which a patient with a PVA positive for HIT antibodies was successfully treated with PVA resection and anticoagulation therapy with argatroban.

## Introduction

Popliteal venous aneurysms are rare and are associated with dilated lesions of peripheral veins.^1)^ Most are asymptomatic, but when complicated by acute pulmonary embolism, they can be fatal and require immediate surgical intervention.^2)^ The treatment for acute pulmonary embolism often involves anticoagulation therapy with heparin. Heparin-induced thrombocytopenia (HIT) antibodies are a serious and potentially fatal complication occurring in 5% of patients exposed to heparin, requiring discontinuation of the causative agent and initiation of alternative anticoagulation therapy.^3)^ In this report, we describe the successful surgical treatment of a giant popliteal venous aneurysm positive for HIT antibodies in a patient with recurrent acute pulmonary embolism.

## Case Report

**Figure 1:**
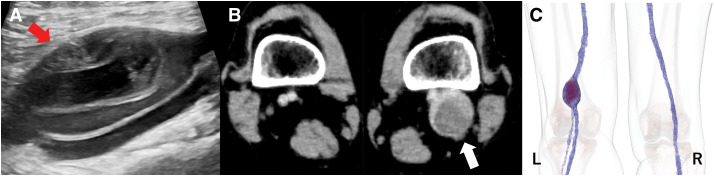
Fig. 1 (**A**) Echocardiography of the veins in the lower extremities revealed a venous aneurysm in the left popliteal fossa (red arrow). (**B**) Contrast-enhanced CT scan showed that the venous aneurysm was 60 mm in diameter and filled with thrombus (white arrow). (**C**) Three-dimensional CT image showed a fusiform-type popliteal venous aneurysm. The image is a dorsal view of the PVA, with the thrombus in the PVA shown in dark purple.

A 56-year-old woman was referred for the treatment of a left popliteal venous aneurysm, 60 mm in diameter, with a massive thrombus inside, which was diagnosed by echography and computed tomography (CT) (**Fig. 1**). She had a history of 2 episodes of acute pulmonary embolisms in 3 months, of which the 2nd attack was complicated by cardiac arrest and was resuscitated with percutaneous cardiopulmonary support (PCPS).

Before referral, she underwent anticoagulation therapy with heparin for acute pulmonary embolism, but developed thrombocytopenia (with her platelet count dropping to less than half) and experienced a right parietal stroke during heparin administration. Her clinical score (4 T’s score)^4)^ was 6 points, and HIT was highly suspected. She was found to be positive for HIT antibodies, confirming the diagnosis of HIT, and her anticoagulation therapy was changed to warfarin.

To prevent the recurrence of embolism from the popliteal venous aneurysm in a patient with HIT, repair of the venous aneurysm was indicated.

**Figure 2:**
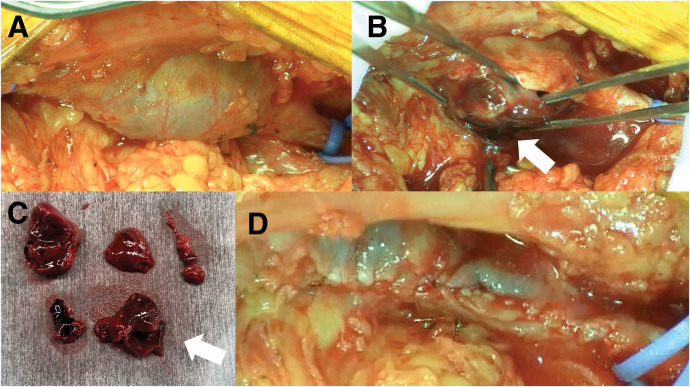
Fig. 2 The popliteal venous aneurysm was dilated (**A**) and filled with a dark red, fresh thrombus (**B**, **C**; white arrow). The redundant venous wall was resected, and a longitudinal running suture with 5-0 polypropylene was used to repair the venous tract to a diameter of 7 mm (**D**).

Under general anesthesia in the prone position, a longitudinal incision was made in the left popliteal fossa, and the popliteal vein was exposed. After the administration of 25 mg argatroban (Argatroban injection [sawai]; Sawai Pharmaceutical, Osaka, Japan), a prolonged activated clotting time (ACT) of at least >200 seconds was confirmed, and the popliteal vein was clamped. The venous aneurysm was opened longitudinally, and dark red fresh thrombus was removed (**Fig. 2**). A part of the thrombus was found to be firmly adherent to the vein wall. The redundant venous wall was resected, and a longitudinal running suture with 5-0 polypropylene was used to repair the venous tract to a diameter of 7 mm. Intraoperative echocardiography confirmed that there was no stenosis and no residual thrombus inside the popliteal vein.

**Figure 3:**
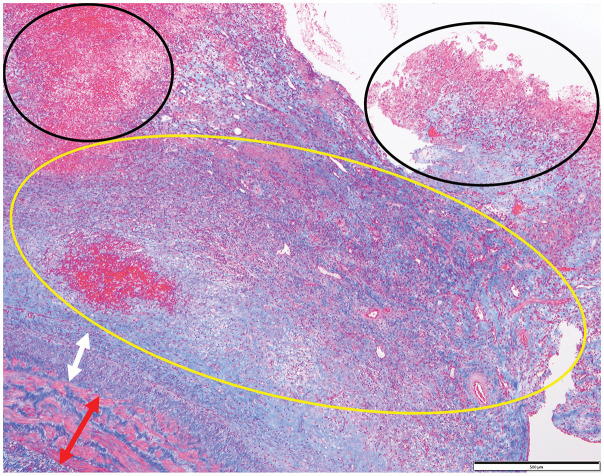
Fig. 3 A thickened tunica media (white arrow) is observed inside the intima (red arrow), and a thrombus adherence (black circle) is observed on the luminal side. Between the fresh thrombus (black circle) and the thickened tunica media, there is evidence of organization with neovascularization (yellow circle).

Postoperatively, echography and contrast-enhanced CT again showed a patent popliteal vein measuring 7 mm in diameter. Her postoperative course was uneventful, and she was discharged home 12 days after surgery. Pathological findings of venous aneurysm wall included irregular areas of thinning and thickening, and the wall structure being indistinct in the thinned areas (**Fig. 3**). There were no findings indicative of phlebitis or malignancy. At 1 year and 3 months postoperatively, no signs of stenosis or occlusion of the repaired left popliteal vein have been observed, and no recurrence of embolism has been detected with the use of the direct oral anticoagulant rivaroxaban (Xarelto tablets 15 mg; Bayer, Osaka, Japan).

## Discussion

Popliteal venous aneurysms are rare,^5)^ and the etiology of progressive dilatation of the vein due to fragility of the vessel wall is suspected to be congenital, traumatic, inflammatory, or due to local degeneration.^6)^ In our present case, pathological examination did not reveal inflammatory changes and only suggested vulnerability of the vessel wall.

Popliteal venous aneurysms are usually asymptomatic; however, acute pulmonary embolism, if complicated, can lead to a life-threatening condition.^1)^ In this case, the patient experienced 2 episodes of pulmonary embolisms, one of which needed resuscitation with PCPS.

HIT antibodies usually become negative within 5–7 days,^7)^ but in this patient positive HIT persisted. To prevent the recurrence of fatal pulmonary embolism, repair of the venous aneurysm was indicated.

Argatroban is a very useful anticoagulation option in HIT antibody-positive patients. Argatroban is a direct thrombin inhibitor and acts independently of heparin, making it safe to use even in HIT antibody-positive patients.^8)^ In addition, argatroban is characterized by a rapid onset of action and a short half-life, providing flexibility in intraoperative and postoperative coagulation management. In the present case, 25 mg of argatroban was administered intraoperatively, and the ACT was prolonged to more than 200 seconds. Postoperatively, the patient was promptly transitioned to warfarin, which was prescribed to maintain a prothrombin time with an international normalized ratio (PT-INR) of around 1.5–2.0. Long-term anticoagulation management was achieved with a direct oral anticoagulant.

Repair of popliteal venous aneurysm is the core of surgical intervention to prevent recurrence of embolism. In this case, after incision of the venous aneurysm and removal of the thrombus, the redundant vein wall was resected and the vein diameter was adjusted with sutures. Although this case involved a fusiform-type PVA, and venous graft replacement is generally performed, there are reports of trimming and plication for fusiform-type PVA.^9,10)^ Considering the long-term patency, we decided to repair the PVA instead of performing venous graft replacement.

The use of intraoperative ultrasound to confirm the presence of residual thrombus or stenosis in the vein ensures successful repair. Ultrasonography is an important tool that allows real-time intraoperative evaluation and reduces the risk of postoperative complications. In addition, pathological evaluation of the venous wall revealed the degree of wall fragility and degeneration, contributing to our understanding of the etiology.

## Conclusion

We report a case in which a patient with 2 episodes of acute pulmonary embolisms due to thrombus in a popliteal venous aneurysm was successfully treated surgically with argatroban for anticoagulation, as the patient was positive for HIT antibody.

## Declarations

### Funding

None.

### Patient consent

Patient consent was obtained.

### Disclosure statement

All authors have no conflict of interest.

### Author contributions

Study conception: ST, KY, HM

Data collection: ST, KY, HM

Analysis: ST, KY, HM

Investigation: ST, KY, HM

Manuscript preparation: ST, KY, HM

Funding acquisition: none

Critical review and revision: all authors

Final approval of the article: all authors

Accountability for all aspects of the work: all authors.
